# Differences in the perceived justice of penalties for road traffic offences between Lithuanian offenders and non-offenders

**DOI:** 10.1371/journal.pone.0269239

**Published:** 2022-06-24

**Authors:** Endriulaitienė Auksė, Justina Slavinskienė, Laura Šeibokaitė, Rasa Markšaitytė, Mark Sullman

**Affiliations:** 1 Vytautas Magnus University, Kaunas, Lithuania; 2 Department of Social Sciences, University of Nicosia, Nicosia, Cyprus; University of Sheffield, UNITED KINGDOM

## Abstract

Most countries around the world use the penalties’ system in order to increase compliance with road traffic rules. However, it can be argued that the most penalties’ systems are ineffective, as drivers do not change offending behavior due to received penalty and repeatedly violate them. The perceived fairness of these penalties may be related to the level of adherence to these traffic rules. Therefore, this research explored the perceived justice of penalties for road traffic rules in a sample of Lithuanian drivers and investigated the differences between offenders and non-offenders. The sample consisted of 358 participants (63.4 percent females, aged 18–75, mean age 35.2 years) who completed an online survey. Participants were asked to report how fair they believed penalties were for road traffic rule offences in general and using a list of 23 specific penalties, including: drink driving, speeding, dangerous maneuverings, illegal overtaking, handheld phone use while driving, etc. The survey measured demographic data, as well as data regarding driving exposure, traffic offences and crashes during the last 12 months. The results revealed that in general drivers perceived the penalties as fair or adequate. The answer “adequate/fair” was chosen most frequently for almost all penalties (from 41.1 to 71.3 percent), with only the penalty for carrying a child without a car seat (€30–50) being perceived as too mild (55.5%). Gender differences were found in the evaluation of the penalty for driving under the influence of alcohol, while age and driving frequency differences were found in the perceived fairness of the penalties for not using a seatbelt, aberrant driving and using a handheld mobile phone while driving. Drivers who reported no offences during the last year, perceived the penalties for speeding as being too mild, when compared to drivers with at least one penalty over the previous year. Contrarily, offenders reported the penalties for speeding as being too severe. Offenders, who experienced driving license suspension, perceived the penalties as being too severe for drink driving and aberrant driving than offenders who received monetary fines. In summary, both Lithuanian offenders and non-offenders generally perceived the penalties for traffic rule offences as adequate and fair, but individual differences and the experience of traffic sanctions were related to the perceived justice of specific penalties.

## Introduction

Traffic law enforcement is widely acknowledged as being a crucial element of traffic safety [1]. Different countries have designed their penalty systems for traffic violations based upon their political, cultural, legal and public health needs. However, research shows that penalties for traffic violations do not deter drivers from unsafe road behavior, as they often repeatedly violate the rules [2]. However, research has shown that more severe penalties are more effective in increasing compliance with road traffic rules [3–5]. Furthermore, research has found the crash risk to be 2–3 times higher for those who have been penalized for dangerous driving or driving under the influence of alcohol [6], and that intentions to comply with the traffic safety rules do not increase after they have been penalized [7]. The repeated violations of road traffic rules result in higher traffic injury and fatality rates all over the world [8]. Despite legal improvements and other preventive actions, traffic accidents due to road traffic rules violations remain a serious public health concern and require further investigation.

There have been a number of studies looking at the reasons drivers disobey road traffic rules, despite knowing the negative potential consequences, such as crashes or sanctions. Several researchers have argued that the perception of enforcement, not the penalty or fine itself, might help to explain the non-adherence to traffic rules, especially in relation to the perceived fairness of the penalty [1, 9]. If the driver perceives the punishment as too severe and not commensurate with the offence, it will be considered to be unjust or unfair [10]. Consequently, stricter penalties are perceived as less fair [11] and drivers react with anger and are reluctant to comply [12–14]. However, the evidence about the relationship between the perceived justice of the penalty and the degree of deterrence remains contradictory, and the factors related to attitudes and perception of justice are under-explored in the literature. Moreover, evidence shows that attitudes towards penalties may change over time. Therefore, it is reasonable to expect that a deeper understanding of individual differences in drivers’ perceptions of penalties might help to develop effective intervention strategies which target the road safety beliefs of the riskiest groups [15].

Although there is some research about drivers’ perceptions of penalties in several countries. However, Alonso et al. [9] emphasizes the need for country-specific research due to findings that legal compliance and the perceived justice of penalties are culturally dependent. Also, they are related to existing formal/ informal social norms or enforcement strategies. Therefore, the application of results between countries is limited or perhaps even impossible. The present study was conducted in Lithuania, an Eastern European country where changes in the traffic law enforcement are regularly implemented. Despite positive progress in decreasing the accident rates since 2015, there remains a high number in traffic injuries, collisions (66 for 100 000 inhabitants per year) and an increasing number of traffic rule violations (343525 in May 2021, compared with 277641 in May 2020) [16]. These results imply that the traffic penalty system is not effective, since it is not preventing on-road risk taking. Traffic safety research in Lithuania has been mostly focused on road infrastructure [17] and measuring aspects of personality which are important for risky driving [18, 19], while there are very few studies which have explored the relationship between attitudes towards penalties and compliance with the law. The present study intended to fill this gap and to explore differences in the perceived justice of penalties for road traffic offences between Lithuanian offenders and non-offenders. In Lithuania traffic violations are sanctioned in three ways–monetary fines, driver license suspension for a certain period of time or prosecution via the criminal justice system (criminal liability and confiscation of vehicle). These may be combined, depending on the severity of the violation, which may cause different perceptions in the level of justice [20].

The theory of Deterrence proposes that there are several components to the perceptions of penalties, such as the perceived certainty of apprehension, perceived severity of punishment, and perceived swiftness of punishment [1, 21–23]. But the present study focuses only on the perceived severity, as this has been found to be the strongest indicator of the perceived justice of traffic law enforcement [10]. The perceived justice of a penalty for violating a traffic rule is usually described as the driver’s belief that the penalty is adequate and concordant with the severity of the violating behavior [1, 24, 25]. As perceived justice might be the antecedent of later on-road violating behavior, it is clearly important to explore this variable in more detail and to analyze driver differences as a potential predictor of each specific belief.

Several authors have investigated individual differences in the perceived justice of sanction in a number of different countries. In general, evidence shows that the majority of non-professional drivers perceive the sanctions for different traffic offences as being fair [9], although the severity is reported as being high [26]. The ESRA-2 project data revealed that the majority of the respondents in all regions agreed that traffic rules should be stricter with regards to driving under the influence of alcohol, speeding and using a handheld mobile phone while driving, while a minority reported that the penalties were too severe and presumably also unfair [27]. However, Rosenbloom & Shahar [28] found that taxi drivers in Israel evaluated penalties as less adequate or unfair when compared to male non-professional drivers. Furthermore, Zámečník et al. [29] conducted a qualitative study and found that DUI drivers thought that the punishment of suspending their drivers’ license was not fair and not applied equally to all offenders in the Czech Republic. Woods [30] reported that ethnic minorities (i.e., Black and Latin inhabitants) and those from lower socio-economic groups in the USA had more negative attitudes towards law enforcement, including traffic enforcement, in comparison to White residents and those from higher socio-economic groups, due to perceived lower justice. In addition, Watling & Leal [14] found that approval of the enforcement of the different types of traffic violations differed significantly, with the highest level of approval being for (in order): DUI enforcement, seat belt use, speeding and driving while fatigued. Moreover, Goldenbeld et al. [11] reported that young males perceived the penalties for speeding as being less fair than the penalties for other road traffic rules violations. In summary, the type of road traffic violation and the different penalty type might foster different levels of perceived justice.

There have been inconsistencies in the reported relationships between several demographic factors and the perceived justice of traffic law enforcement. For example, Goldenbeld et al. [11] reported that gender, age and having been penalized was not related to the perceptions of fairness. Conversely, Freeman et al. [31] reported that women perceived higher severity of punishment for speeding than men did. Goldenbeld & Buttler [27] concluded, from the ESRA-2 data, that women showed a preference for stricter traffic law enforcement than men. Furthermore, young drivers are more likely to report that the punishments for using a handheld mobile phone while driving or driving while fatigued were too severe and less fair, when compared with older drivers [14, 27].

In summary, it appears that studies have more frequently investigated the justice beliefs about punishments for one or several specific violations, while a more general approach to the perceptions of different rule violations has been rare. In addition, very little research has explored differences in the justice beliefs between road traffic rule offenders and non-offenders. Finally, previous research has mostly focused on measuring justice in terms of being asked to evaluate the severity or justice of the penalty. However, a general evaluation of penalties as being “unfair” does not provide the full picture, since a penalty may be perceived as being too mild or too severe. In both cases participants would rate the penalties as being unfair, but the actual meaning has extremely different implications for improving perceptions of justice. Therefore, the present explorative study contributes to the literature new information using a more extensive list of penalties and traffic rule violations, as well as comparing the perceptions of offenders and non-offenders. The study tested the hypothesis that offenders perceive traffic sanctions to be less fair in comparison to non-offenders. This hypothesis is based on the previous literature which shows that those who are considered to be safe drivers, rather than unsafe drivers, have more positive attitudes to different interventions or safety precautions [27, 32], and therefore we expect this pattern to appear for the perceptions of justice.

## Method

### Participants

Three hundred and fifty-eight drivers participated in an online survey. The inclusion criterion was solely the possession of a valid driver license. Demographic information of the sample is presented in Table 1. Their age ranged from 18 to 75 years old, with a mean age of 35.2 years old (SD = 11.1). The level of driving experience ranged from several months to 57 years, with a mean or 14.2 years (SD = 9.8). The majority of the sample drove every day or several times per week (N = 335), while the remainder drove at least once a month (N = 23). Thirty-six study participants were novice drivers (driving experience less than two years), five participants were professional drivers, and the remainder were experienced non-professional drivers.

**Table 1:** Sociodemographic information of study sample.

Variable	Study sample: Lithuanian drivers
Size	358
Males	131
Females	227
Age in years	
mean	35.2
SD	11.1
Driving experience in years	
mean	14.2
SD	9.8
Driving frequency	
less than once per week	23
at least once per week	46
daily driving	289
Penalties during the last 12 months	
no (non-offenders)	259
yes (offenders)	99

Two hundred fifty-nine participants (87 males and 172 females) reported no penalties during the last 12 months and were classified as non-offenders, whereas 99 participants (44 males and 55 females) reported one or more than five penalties in the previous year, with this group being classified as offenders. No statistically significant gender (χ^2^ (358) = 3.18, p = .74), age (*U* (358) = 12.637.000, p = .83), education (χ^2^ (358) = 1.59, p = .81) or driving experience (χ^2^ (358) = 1.90, p = .16) differences were found between the offender and non-offender drivers’ groups. Twenty-three drivers (15 males and 8 females) from the offenders group reported that they had a suspended license, due to the following traffic violations: 34.8% for speeding, 21.7% drove under the influence of alcohol and the remainder were for dangerous driving manoeuvres.

### Instruments

Data were collected using a self-report questionnaire that was developed following the procedure specified by Rosenbloom & Shahar [28]. Participants were provided with a list of 23 penalties for different traffic rule violations and were asked to evaluate how fair each of them was. The list included the specific traffic violation and the three types of possible penalties (monetary fines, license suspension and legal prosecution/ court decisions) following the legislation used in Lithuania (see Table 2). A three-point scale was used to evaluate the perceived justice of each penalty (1 –“too severe”, 2 –“fair”, 3 –“too mild”). Thus, two types of penalties evaluations (“too severe” and “too mild”) were considered to be unfair. The Cronbach alpha for the scale was .93. The results of the scale were calculated using the mean score (the closer the score to 2, the fairer the penalty was perceived to be; the closer the mean to 1, the more severe the penalty was perceived to be; the closer the mean to 3, the milder the penalty was perceived to be).

**Table 2:** The list of items that were included in the questionnaire.

Traffic violation	Penalty
Driving under the influence of alcohol when BAC was less than .4 (novice drivers)	Monetary fine from €230–300
Driving under the influence of alcohol when BAC is .41–1.5	Monetary fine from €800–1100 and license suspension from 12 to 18 months
Driving under the influence of alcohol when BAC is more than 1.5	Monetary fine from €579–868 and license suspension from 24 to 36 months or legal prosecution
Driving 21–30 KPH above speed limit	Monetary fine from €30–90
Driving 31–40 KPH above speed limit	Monetary fine from €120 to 170
Driving 41–50 KPH above speed limit	Monetary fine from €170 to 230
Driving over 50 KPH above speed limit	Monetary fine from €450–550 and license suspension from 1 to 6 months
Dangerous overtaking (against the rules)	Monetary fine from €170–230 and license suspension from 3 to 6 months
Dangerous driving maneuver (when other traffic participants have to brake suddenly, or take other actions in order to ensure safety)	Monetary fine from €170–230 and license suspension from 3 to 6 months
Aberrant driving, intentional dangerous driving which threatens the safety of other traffic participants	Monetary fine from €450–550 and license suspension from 12 to 24 months
Using a handheld mobile phone	Monetary fine from €60–90 and/or license suspension from 1 to 3 months
Driving on outer road margins	Monetary fine from €60–90 and license suspension from 1 to 3 months
Disobeying a sign or road marks	Monetary fine from €60–90 and license suspension from 1 to 3 months
Disobeying a no-entrance sign	Monetary fine from €60–90 and license suspension from 1 to 3 months
Driving without lights on (as required)	Monetary fine from €30–40
Entering a junction during a red light	Monetary fine from €60–90
Not stopping before a railway	Monetary fine from €180–280 and license suspension from 2 to 6 months
Not giving way at a junction to vehicles with priority	Monetary fine from €60–90
Not stopping at a stop sign	Monetary fine from €30–90
Not giving way on a roundabout	Monetary fine from €60–90
Passenger without a seatbelt	Monetary fine from €30–50
Child not restrained	Monetary fine from €30–50
Driver without a seatbelt	Monetary fine from €30–50

*BAC refers to Blood Alcohol Content

** KPH refers to kilometers per hour

In order to look for higher level constructs in the perceived justice of the different penalties, an exploratory factor analysis was conducted. The principal component analysis (PCA) method was used with Varimax rotation. The KMO index (.914) indicated that the data was appropriate for factor analysis, and the Bartlett’s Test of Sphericity was significant (χ^2^ (253) = 4329.17, p < .0001), indicating that the relationships among the variables were strong and the data were suitable for conducting an Exploratory Factor Analysis. Using Parallel Analysis (PA), five factors and one individual item were obtained, which had Eigenvalues > 1. The coefficients for items were displayed by size (see Table 3), with correlations < .40 being suppressed, and the extracted factors explained a total variance of 68.32%. The five factors were named as follows: Factor 1: Penalties for Driving under the influence of alcohol (three items); Factor 2: Penalties for Speeding (four items); Factor 3: Penalties for Aberrant driving (three items); Factor 4: Penalties for Seatbelt non-use (three items); Factor 5: Penalties for Other traffic violations (nine items). One factor was formed from only one item ‘Penalty for Using mobile phone while driving’. Therefore, this would not be considered a factor, the single item will be use in further analysis.

**Table 3:** Factor loadings by EFA (pattern matrix of the factors and items).

Pattern matrix						
Penalties for:	Component					
	1	2	3	4	5	6
Driving under the influence of alcohol when BAC is less than .4 (novice drivers)	.692					
Driving under the influence of alcohol when BAC is .41–1.5	.808					
Driving under the influence of alcohol when BAC is more than 1.5	.751					
Driving 21–30 KPH above speed limit		.767				
Driving 31–40 KPH above speed limit		.795				
Driving 41–50 KPH above speed limit		.816				
Driving over 50 KPH above speed limit		.698				
Dangerous overtaking			.688			
Dangerous driving manoeuvre (other traffic participants have to suddenly brake, change lane or take other actions to avoid a collision)			.782			
Aberrant driving, intentional dangerous driving with the threat to the safety of other traffic participants			.742			
Using a handheld mobile phone				.436		
Driver without a seatbelt					.837	
Passenger without a seatbelt					.826	
Child not in a child seat					.627	
Not giving way on a roundabout						.807
Not giving way at a junction for vehicles with priority						.788
Not stopping at a stop sign						.780
Disobeying a sign or road markings						.717
Disobeying a no-entrance sign						.708
Not stopping before a railway						.665
Driving without lights (when required)						.613
Driving on outer road margins						.540
Entering a junction during a red light						.437

*BAC refers to Blood Alcohol Content

** KPH refers to kilometers per hour

Reliability of the factors were tested using Cronbach alpha, with all five factors having a score above .70 (Factor 1 Cronbach alpha–.73, Factor 2 –.88, Factor 3 –.79, Factor 5 –.82, Factor 6 –.87). The Cronbach alpha for the one item (penalty for mobile phone use) could not be calculated.

The scores for each factor were calculated as a mean number of participants’ agreements with the statements that the penalty was “too mild”, “too severe” and “fair/adequate”. Therefore, higher proportions in each justice evaluation category (too mild, too severe or fair/adequate) indicates stronger agreement with one of the three evaluation categories.

## Procedure

Data was collected using an online survey and convenience sampling. Participants were invited to the study via social media and those who agreed to participate were offered no reward for taking part. Informed consent was obtained by assuring participants of the anonymity and confidentiality of all data they provided, that their participation was completely voluntary and that no individual data would be published.

All data analyses were performed using IBM SPSS–version 23.0, including the descriptive statistics and all inferential statistics. Comparison of the perceived justice of penalties for violating road traffic rules were conducted using χ^2^ analysis and the non-parametric Mann-Whitney test, due to the non-normality of the distributions. A Wilcoxon Signed Ranks Test was used to compare the means of the factors described previously (see Table 4).

**Table 4:** The descriptive statistics of penalty’s perceived justice scores in a driver sample (N = 358).

Traffic violation	Penalty	Total sample (N = 358)				
		Mean scores	SD	Evaluation of justice (%)		
				Too severe	Fair/	Too mild
					Adequate	
**Factor 1: Driving under the influence of alcohol**		2.26	.48			
Driving under the influence of alcohol (BAC is less than .4 for novice drivers)	Monetary fine from €230 to 300	2.25	.58	7.3	60.3	32.4
Driving under the influence of alcohol (BAC is .41–1.5)	Monetary fine from €800–1100 and license suspension from 12 to 18 months	2.11	.61	14.0	61.5	24.6
Driving under the influence of alcohol (BAC is more than 1.5)	Monetary fine from €579–868 and license suspension from 24 to 36 months or legal prosecution	2.41	.59	5.0	48.6	46.4
**Factor 2: Speeding**		2.20	.54			
Driving 21–30 KPH above speed limit	Monetary fine from €30–90	2.12	.61	13.1	61.5	25.4
Driving 31–40 KPH above speed limit	Monetary fine from €120–170	2.14	.63	13.7	58.7	27.7
Driving 41–50 KPH above speed limit	Monetary fine from €170–230	2.29	.65	10.9	49.2	39.9
Driving over 50 KPH above speed limit	Monetary fine from €450–550 and license suspension from 1 to 6 months	2.25	.64	11.2	52.5	36.3
**Factor 3: Aberrant driving**		2.12	.56			
Dangerous overtaking	Monetary fine from €170–230 EUR and license suspension from 3 to 6 months	2.05	.63	20.1	46.9	33.0
Dangerous driving (causing other drivers to brake suddenly, swerve or take other actions in order to avoid a crash)	Monetary fine from €170–230 EUR and license suspension from 3 to 6 months	2.19	.66	13.7	53.6	32.7
Aberrant driving, intentional dangerous driving with the threat to the safety of other traffic participants	Monetary fine from €450–550 and license suspension from 12 to 24 months	2.13	.72	17.3	60.3	22.3
**Factor 4: Seatbelt non-use**		2.25	.52			
Driving without a seatbelt	Monetary fine from €30–50	2.20	.62	11.5	57.5	31.0
Passenger without a seatbelt	Monetary fine from €30–50	2.04	.63	18.2	60.1	21.8
Child not restrained with a seatbelt	Monetary fine from €30–50	2.53	.56	3.4	40.2	56.4
**Factor 5: Other Road signs violations**		1.91	.43			
Not stopping at a stop sign	Monetary fine from €30–90	2.15	.51	6.7	71.8	21.5
Not giving way on a roundabout	Monetary fine from €60–90	2.04	.55	13.4	69.3	17.3
Not giving way at the junction for vehicles with priority		2.14	.58	10.6	64.8	24.6
Driving on outer road margins	Monetary fine from €60–90 and license suspension from 1 to 3 months	1.72	.64	38.0	51.7	10.3
Disobeying a sign or road markings		1.67	.606	40.2	52.5	7.3
Disobeying a no-entrance sign		1.66	.64	42.7	48.0	9.2
Not stopping before a railway	Monetary fine from €180–280 and license suspension from 2 to 6 months	1.81	.59	29.1	60.9	10.1
Entering a junction during a red light	Monetary fine from €60–90	2.31	.62	8.7	51.7	39.7
Driving without lights, when required	Monetary fine from €30–40	1.73	.66	39.1	49.2	11.7
**Single item: Using phone while driving**		1.97	.70			
Using a handheld mobile phone	Monetary fine from €60–90 and/or license suspension from 1 to 3 months	1.97	.70	26.3	50.8	22.9

*BAC refers to Blood Alcohol Content

** KPH refers to kilometers per hour

## Results

The descriptive statistics are presented in Table 4, which shows that in general drivers evaluated penalties as being fair. The answer “adequate/fair” was chosen most frequently for almost all penalties (range 41.1% to 71.3% of the sample). Only the €30–50 penalty for driving a child without a car seat was perceived as too mild (55.5% of participants). It is worth noting that the penalty for driving under the influence of alcohol, when BAC is more than 1.5 (monetary fine €579–868 and license suspension 24–36 months or legal prosecution), split the sample almost equally—48.6% perceived this penalty as being fair/adequate and 46.4% perceived it as being too mild. Even though almost half of the total sample rated the monetary fine (€60–90 and license suspension from 1 to 3 months) for other road sign violations (e.g., driving on outer road margins, disobeying a sign, road marks or a no-entrance sign) as being fair/adequate, a similar proportion rated this penalty as being too severe. The same tendency is seen for the monetary fine (€60–90) for driving without lights, when they are required.

A Wilcoxon Signed Ranks Test found that the perceived justice of the penalty for driving under the influence of alcohol was the most fair/adequate, when compared with penalties for speeding (Z = -2.54, p = .01), aberrant driving (Z = -2.73, p = .006), using a handheld phone while driving (Z = -4.78, p < .0001) and other road sign violations (Z = -5.47, p < .001). No significant differences were found when comparing the perceived justice of the penalty for driving under the influence of alcohol with failing to wear a seatbelt (Z = -.123, p = .90).

There were no statistically significant gender differences found in the perceived justice of the penalties for speeding, using a handheld phone while driving, seatbelt non-use and other road signs violations.

**Fig 1:**
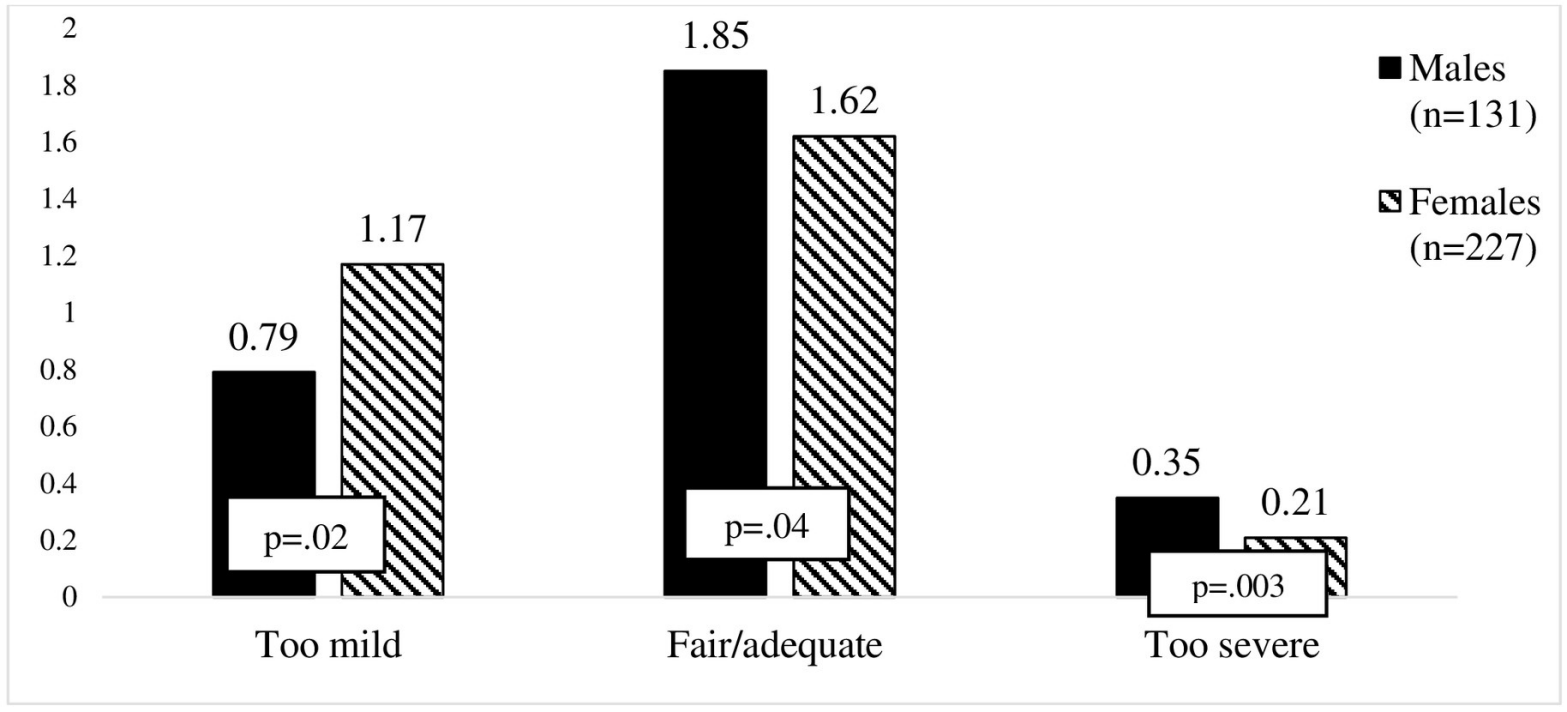
Illustration of differences in evaluation of penalties for driving under the influence of alcohol. *p value is .05. Student t test was used in analysis.

**Fig 2:**
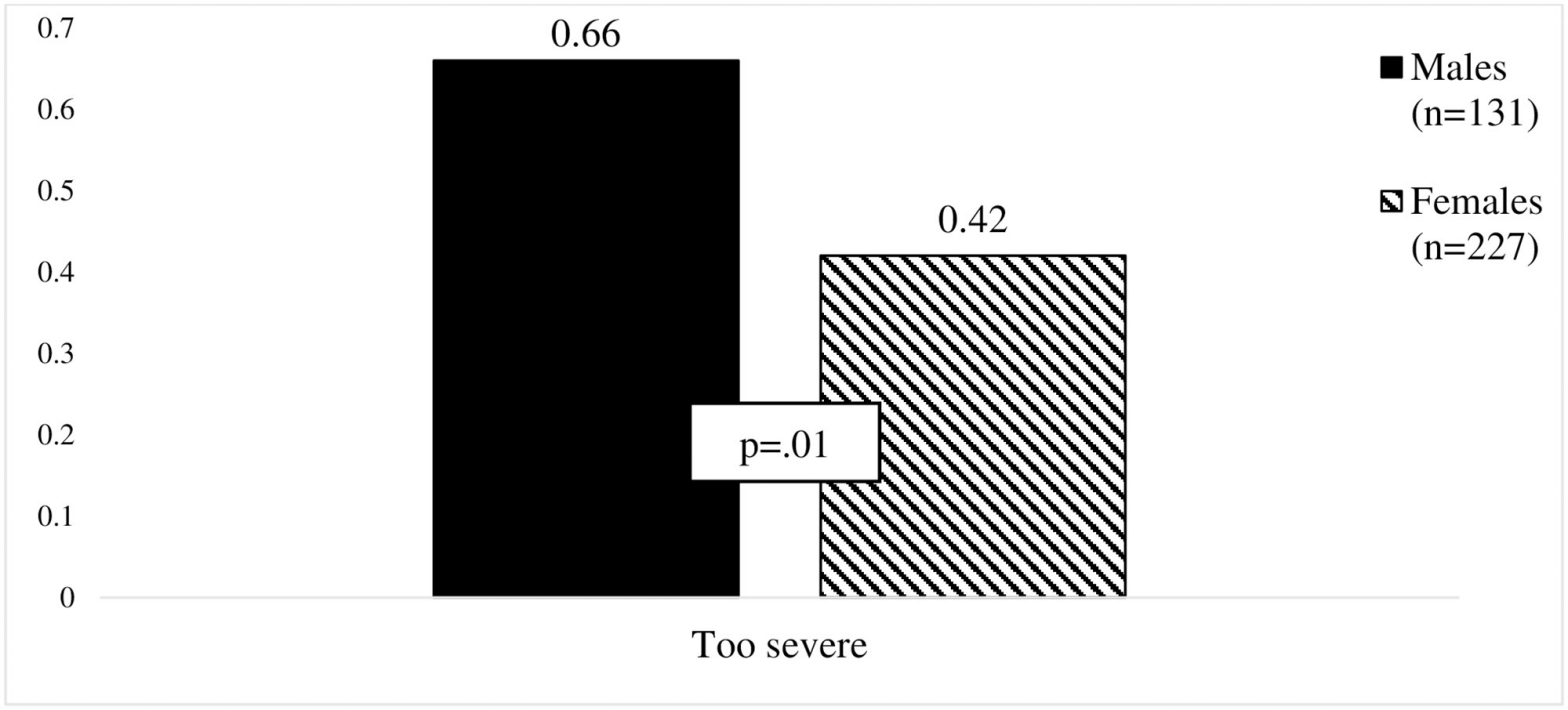
Illustration of differences in evaluation of penalties for aberrant driving. *p value is .05. Student t test was used in analysis.

However, statistically significant differences were found between male and female drivers in the perceived justice beliefs of the punishments for driving under the influence of alcohol (see Fig 1). Males evaluated the penalties for driving under the influence of alcohol as fair/adequate more frequently than females. The same pattern was also seen for the perceived justice of the penalties for driving under the influence of alcohol evaluating them as too severe. In comparison, females, but not males, evaluated the penalties for driving under the influence of alcohol as too mild. In contrast, only males evaluated the penalties for aberrant driving as too severe (see Fig 2).

Further, participants were divided into two groups according to the mean age of the total sample (35.2 years). One hundred and ninety-two participants were 35 and younger, with 166 being 36 years and older. The comparison of the perceived justice of the penalties showed no statistically significant differences by age, except one. Younger drivers evaluated the penalties for seatbelt non-use as being too mild (Mean = 1.23, SD = 1.1), when compared to older drivers (Mean = .93, SD = 1.1) (*U*_too mild_(358) = 13.578.000, p = .01).

Those drivers who rated their driving frequency as “once a month” or “less than once a week” were grouped together and they were classified as drivers with lower driving exposure. This group was the minority (9.2%) in the total sample, with the remainder classified as frequent drivers. There were no statistically significant differences in the perceived justice of penalties, by driving exposure, for driving under the influence of alcohol, speeding, seatbelt non-use and violations of other road signs. However, significant differences were found for aberrant driving behaviors and using a handheld phone while driving. Drivers with lower driving exposure perceived the penalties for aberrant driving as being too mild (Mean = 1.39, SD = 1.2) more often than drivers who drove on a daily basis (Mean = .84, SD = 1.1), (*U*_too mild_ (358) = 2,878.000, p = .02). Drivers with high driving exposure evaluated the penalties for using a handheld phone while driving as too severe (mean = .27, SD = .4) more often than those with lower driving exposure (mean = .09, SD = .2; *U*_too severe_ (358) = 4,575.500, p = .04).

In order to test the hypothesis that offenders were more likely to perceive traffic penalties as unjust, when compared to non-offenders, the two groups were compared using the Mann Whitney test. Firstly, the overall scores of the scales were compared for the two groups. A higher number in each justice evaluation category (too mild, too severe or fair/adequate) indicated stronger acceptance of each evaluation category. Results (see Table 5) show no significant differences between the offender and non-offender groups were found in the overall perceived justice of the penalties for traffic violation.

**Table 5:** Comparison of the perceived penalties justice in general scale between offenders and non-offenders.

	Drivers’ group	Too mild		Fair/adequate		Too severe	
		Mean	SD	Mean	SD	Mean	SD
Overall perceived justice of the penalties for traffic violations	Non-offenders (n = 259)	5.32	4.8	12.89	4.8	4.79	4.6
	Offenders (n = 99)	6.32	5.7	12.79	4.8	3.89	4.3
Statistical significance of model		11,733.500, p = .21		13,057.500,		14,338.000,	
				p = .78		p = .08	

**Fig 3:**
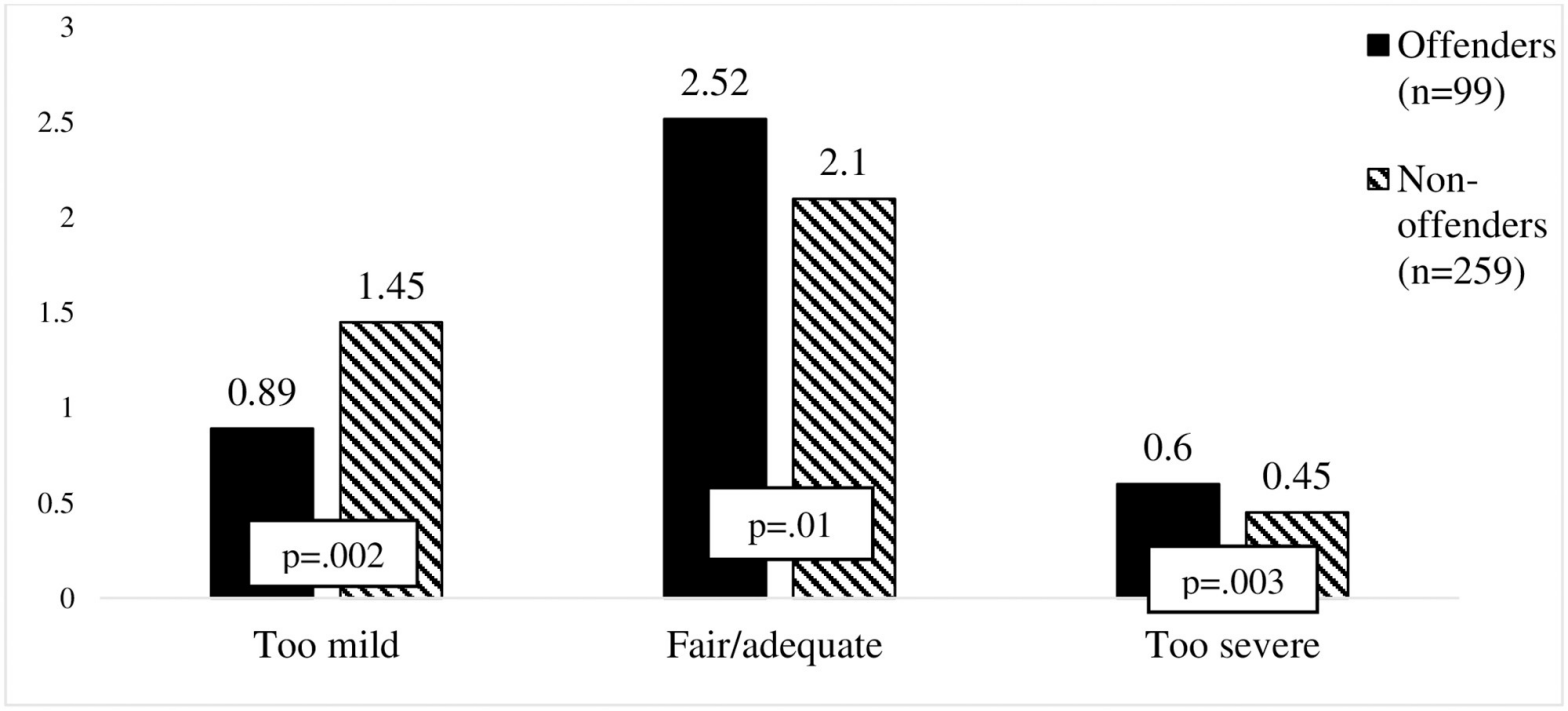
Illustration of differences in evaluation of penalties for speeding among offenders and non-offenders.

Further, the perceived justice of each road traffic rules violation, presented in factors and with single items, were tested taking into account offending status. There were no statistically significant differences found in the perceived justice of the penalties, between offenders and non-offenders, for driving under the influence of alcohol, aberrant driving, using a handheld phone while driving, seatbelt non-use and other road signs violations. However, the results (see Fig 3) indicated that less safe drivers (offenders) perceived the penalties for speeding more frequently as fair/adequate (*U*_fair/adequate_ (358) = 14,818.500, p = .01) than did non-offenders. Also, less safe drivers (offenders) more often perceived the penalties for speeding as too severe (*U*_too severe_ (358) = 14,790.500, p = .003), compared with those who had no offence during the last year. However, safer drivers (non-offenders) evaluated the penalties for speeding as too mild (*U*_too mild_ (358) = 10,271.000, p = .002) more often than offenders.

As the perceived justice of the penalties for speeding differed significantly between offenders and non-offenders, this factor was analyzed in more detail, looking at the specific penalties for the different types of speeding offences (see Table 6).

**Table 6:** The perceived justice of the penalties for the types of speeding offences (offenders vs. non-offenders).

Factor 2: Speeding			Evaluation of justice (%)			
Traffic violation	Penalty	Group	Too severe	Fair/	Too mild	Statistical significance of model
				Adequate		
Driving 21–30 KPH above speed limit	Monetary fine €30–90	Non-offenders (n = 259)	11.2	61.0	27.8	χ^2^(2) = 4.78, p = .09
		Offenders (n = 99)	18.2	62.6	19.2	
Driving 31–40 KPH above speed limit	Monetary fine from €120–170	Non-offenders (n = 259)	12.7	55.2	32.0	**χ** ^**2**^ **(2) = 9.04 p = .01**
		Offenders (n = 99)	16.2	67.7	16.2	
Driving 41–50 KPH above speed limit	Monetary fine from €170–230	Non-offenders (n = 259)	10.4	45.2	44.4	**χ** ^**2**^ **(2) = 7.87, p = .02**
		Offenders (n = 99)	12.1	59.6	28.3	
Driving over 50 KPH above speed limit	Monetary fine from €450–550 and licence suspension 1–6 months	Non-offenders (n = 259)	10.4	49.0	40.5	**χ** ^**2**^ **(2) = 7.23, p = .03**
		Offenders (n = 99)	13.1	61.6	25.3	

NOTE: bolded results indicate statistically significant differences in the perceived penalties for speeding between non-offender and offender drivers.

Table 6 shows that a statistically significantly higher percent of offenders perceived three types of penalties for speeding as too severe, when compared with non-offenders. The exception was the penalty for driving 21–30 KPH above the speed limit (p = .09). In addition, significantly more non-offenders perceived the penalties for speeding as too mild, again except for the same monetary fine from €30–90 (p = .09). At least half of the offenders and non-offenders reported the penalties for speeding to be fair and adequate.

Previous studies have found that the more severe a penalty is for violating road traffic rules, the more unfair it is evaluated (Goldenbeld & Buttler, 2020). Therefore, the present study aimed to evaluate whether traffic offenders who experienced a suspension of their driving licence evaluated penalties differently from those who violated traffic rules, but only received a monetary fine. This comparison revealed no statistically significant difference in the perceived justice between the two groups using the total score (summed scale). Furthermore, no statistically significant differences were found for the evaluation of the penalties for speeding, using a handheld phone while driving, seatbelt non-use and other road signs violations (those who temporally lost their driving licence vs. those who only received a monetary fine).

**Fig 4:**
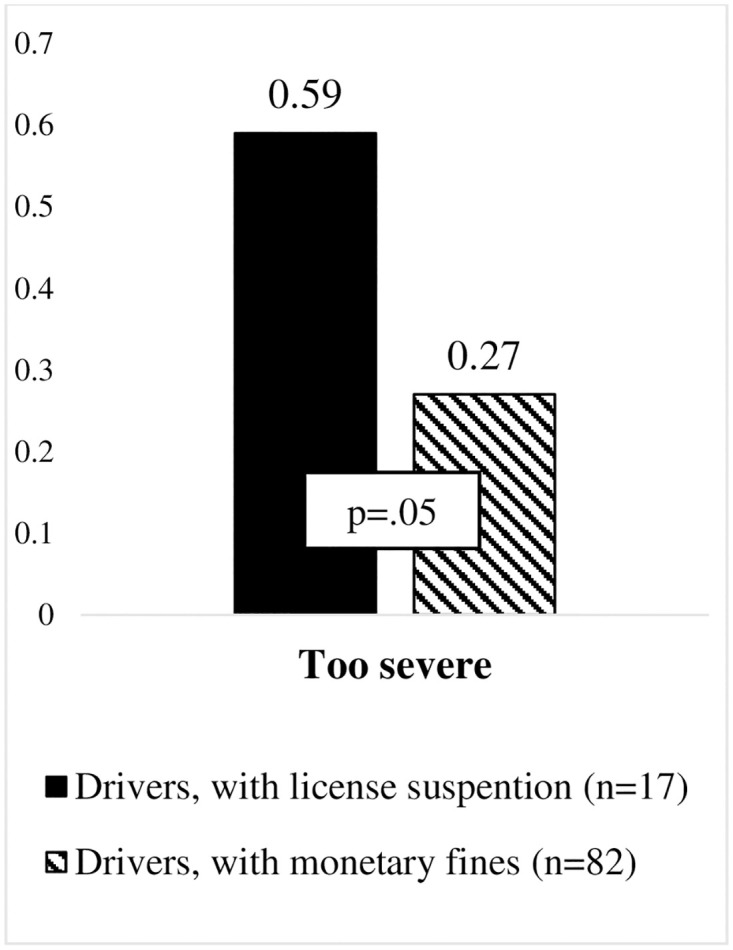
Illustration of differences in evaluation of penalties for DUI among drivers with license suspension and monetary fines.

**Fig 5:**
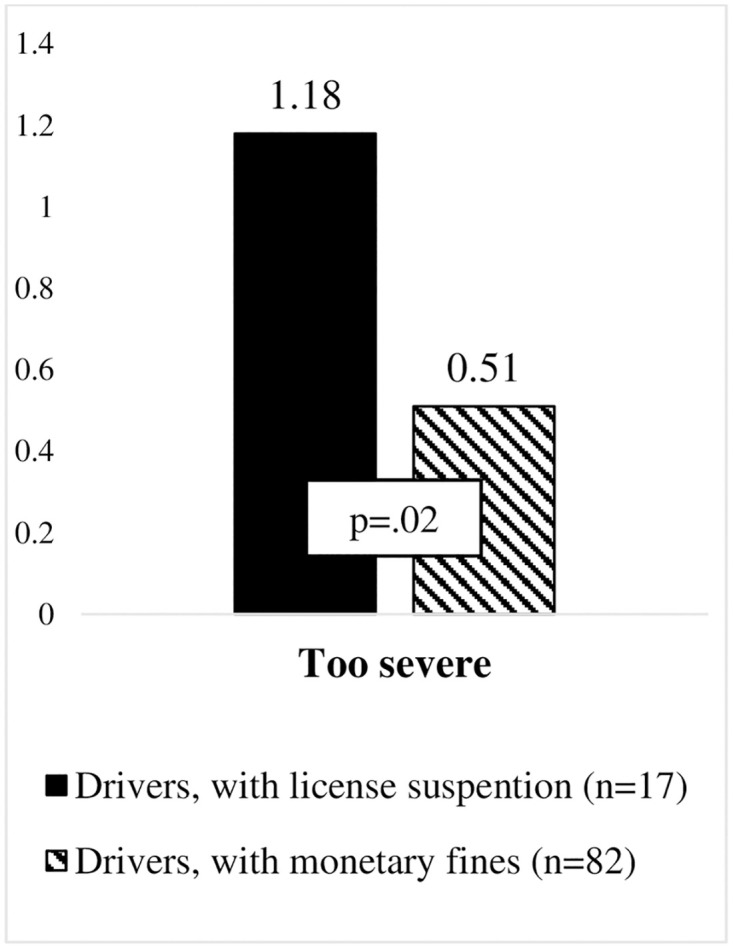
Illustration of differences in evaluation of penalties for abberant driving among drivers with license suspension and monetary fines.

However, offenders who experienced a licence suspension perceived the penalty for driving under the influence of alcohol (see Fig 4) (U_too severe_(99) = 853.000, p = .05) and the penalty for aberrant driving (see Fig 5) (U_too severe_(99) = 896.000, p = .02) as too severe, when compared to those who only received a monetary fine.

## Discussion

The main objective of the current study was to explore the perceived justice of the penalties for 23 road traffic rules violations in a sample of Lithuanian drivers and to investigate differences among offenders and non-offenders. The results showed that most drivers perceived existing penalties for road traffic rules violations as fair and appropriate, which replicates the results from previous studies [9, 27, 28]. The range in agreement that the specific punishments were fair ranged from 40% to 72%, with the largest agreement being for the penalty “not stopping at a stop sign” and the lowest agreement was for the penalty for transporting a “child not restrained in a child seat”. Interestingly, the largest proportion of drivers perceived the penalty for “child not restrained in a child seat” as being too mild (56% of the sample), rather than fair (40% of the sample). Furthermore, a deeper analysis of the results revealed that more drivers agreed that the punishments for driving under the influence of alcohol, speeding, aberrant driving and seatbelt non-use were fair or too mild, while the penalties for other rule violations were fair or too severe (except for the violation “entering a junction during a red light”). This might be due to the fact that driving under the influence of alcohol and speeding are the two largest causes of crashes all over the world, and seatbelt non-use is regularly communicated in the media as the cause of fatalities in crashes, so these infringements are perceived in society as being the riskiest and more immoral road behaviors [14, 33]. In contrast, other violations that are not as severely penalized, in terms of the financial penalty, might be perceived as more acceptable by society and less dangerous in nature. The importance of non-legal sanctions, as well as the emotional reactions to the potential outcomes of severe violations (like fear of personal loss), mean that it is not only societal norms which contribute to the different evaluations of justice [1, 34]. Furthermore, Rosenbloom & Shahar [28] argue that less severe offences are more frequently committed, so drivers may foresee a higher probability of receiving sanctions for these offences, which fosters the perceptions of injustice or the negative evaluation (as too severe) of these penalties in advance.

Significant gender differences were found in the perceived justice of the penalties for drink driving and aberrant driving. Similarly, in line with the findings of Watling & Leal [14], the present study found that males more often perceived the penalties for driving under the influence of alcohol as being fair/adequate than did females. Also, men more frequently evaluated these penalties as being too severe, whereas women reported them to be too mild more frequently than men. These results are partially in line with those of Goldenbeld et al. [11] and Zámečník et al. [29], who both found that males perceived the penalties for driving under the influence of alcohol as being too severe. This might be explained by the findings that males have more positive attitudes toward driving under the influence of alcohol [35]. Previous research has also found that males perceive driving under the influence of alcohol and other risky driving behaviors as less dangerous than females [36]. Therefore, the penalties for drink driving and for aberrant driving were evaluated as too severe, because these evaluations are not based on their perceptions of risk or the potential severity of the consequences of these behaviors [9]. It is important to note that more females, than males, perceived the penalties for driving under the influence of alcohol as being too mild. This confirms previous findings that females tend to drive in a less risky manner and to perceive all dangerous behaviors on the road as serious [37]. Thus, the more serious a behavior is perceived to be, the more severe the expected penalty [31].

The current study also confirmed that age and driving frequency are important factors for evaluating the perceived justice of the penalties of seatbelt non-use and illegal phone use [38]. Our findings suggested that younger drivers more often perceived the penalties for seatbelt non-use as too mild, than did older drivers. This result might be explained using the premise proposed by Bates and Anderson [22], that young and inexperienced drivers have more fears about safety while driving, since they have less driving experience, but they have an increased need for driving safety. Perhaps, due to age and inexperience, younger drivers perceive seatbelt non-use as riskier behavior in comparison to older age drivers, who have experience to drive with not using seatbelt and not being punished for it. Thus, seatbelt non-use is evaluated as being more serious and dangerous and the penalty for this should be severe enough in order to deter a recurrence of this behavior [22]. This explanation is supported by several other findings in the present study. The results of this study also showed that drivers who drove less frequently perceived the penalties for aberrant driving as being too mild more often than did drivers who drove more frequently. It is likely that drivers who rarely drive do not have as high self-confidence as those who drive daily. As a result, they might be more sensitive to aberrant behavior on the road and expect more severe outcomes from this behavior. The present results also support the previous results of Goldenbeld & Buttler [27], as those who drove more frequently perceived the penalties for mobile phone use while driving as not being fair. Up to 75% of road users admit to talking on a hand-held phone while driving and thus this risky behavior could be treated as a habit. Therefore, the majority of the drivers underestimate the risks associated with this behavior and tend to defend themselves by evaluating the penalties for this behavior as being too severe [38].

The results only partially supported the assumption that non offenders (drivers without a penalty in the last 12 months) possess more positive beliefs about the justice of traffic penalties, when compared to drivers who had been penalized in the last 12 months (offenders). The general evaluation of the perceived justice of traffic penalties (when the different sanctions for the different penalties are all combined) did not differ between offenders and non-offenders. This contradicts the results reported by Rosenbloom & Shaban [28], who found that more risky drivers (like taxi drivers) perceived penalties as being less just, when compared to non-professional drivers (assumed to be less risky drivers). Our findings also disagree with those of Zámečník et al. [29], who found that DUI drivers reported that the suspension of their license to drive was unfair. On the other hand, our data support the conclusion of Watling & Lead [14] that when analyzing and interpreting the justice perceptions, specific violations and the penalties for them should be taken into account, rather than using general justice beliefs. The results of the current study showed that the penalties for speeding were more often evaluated as being too severe by the offenders’ group, whereas the non-offenders evaluated these penalties as being too mild. As other penalty evaluations did not differ, it seems that speeding is somehow a unique violation for Lithuanian drivers. Rosebloom & Shaban [28] proposed the explanation suggested by the theory of social bonds, which states that differences in perceived justice may occur because some groups of drivers (especially risk takers) are more prone to follow informal rules rather than formal laws. In Lithuania, speeding violations are so common that the informal acceptance of this behavior among drivers may lead to different justice beliefs, but this hypothesis should be tested in future research. On the other hand, the differences in the perceived justice of penalties for speeding (offenders vs. non-offenders) may confirm the proposition in previous literature that risk averse drivers, rather than risk prone drivers, hold more positive attitudes to different safety precautions [27, 32]. This explanation is also supported by the findings of the current study, which showed that offenders with a license suspension, due to a severe violation, perceived the penalty for driving under the influence of alcohol and the penalty for aberrant driving as being too severe more often than offenders who were only sanctioned with a monetary fine.

The perceived justice of traffic law enforcement is normally related to subsequent violating behavior, and thus examining factors associated with compliance, such as attitudes towards regulations or legitimacy beliefs, have important implications for traffic safety. Drivers’ beliefs about law enforcement have received limited attention in Lithuania and in many other countries [14], so the results of this study contribute to the sparse literature in this area. This study revealed several differences, between offenders and non-offenders, in the perceived justice of penalties for dangerous and relatively common road traffic violations in Lithuania.

The finding that the penalties for speeding, aberrant behaviour and driving under the influence of alcohol are evaluated as unfair by offenders has important implications for understanding the cognitive mechanisms behind serious traffic rule violations. The results of the present study also reveal the need for additional interventions for offenders which focus on changing the perceived justice of these penalties. Given that speeding is currently the most common violation in Lithuania, it is crucially important to change the perception of the speeding behavior itself, as well as the seriousness of the sanctions for this risky behaviour. This approach might help to deter previous offenders from re-offending and increase compliance with the law. Also, although many previous studies have focused on the perceptions of justice or unfairness in general, this study was one of the first to examine the perceptions of penalties in a more balanced way. The evaluation of a penalty as being too mild or too severe provides more insight into how favorably (too mild) or unfavorably (too severe) these penalties are perceived. The results from this perspective allow us to draw the conclusion that some penalties are treated as inadequate among both offenders and non-offenders.

Finally, some limitations of this study should be noted. First of all, convenience sampling was used, meaning that self-selection bias might be an issue, which should be borne in mind when interpreting the results. The sample was also uneven, with an over-representation of non-offenders and a small number of offenders. Although such a proportion might be expected, based on the statistics of the general population, future studies should re-run the study with a more even spread of drivers with different penalty experiences. The use of self-report data might also result in socially desirable responding, as the information about the received penalties might be sensitive in nature and may have affected the results. However, the data were collected using an on-line survey and participants were assured of confidentiality and anonymity, which helps to reduce the impact of this bias [39]. In addition, it should also be noted that there might be some bias in the term “traffic offender” or “non-offender”, as this study used only subjectively reported traffic punishment history over the previous year. This method for grouping drivers is consistently used in the literature, but it might be not precise–as it might be that some drivers in the non-offenders’ group might have engaged in severe violations, similar to offenders’ group, but were not caught by police and were not sanctioned. In that case they report “no penalties in the previous year” and are addressed as non-offenders, while their behavior or beliefs might be more similar to the offenders. Furthermore, the cross–sectional method and the exploratory nature of the study does not allow us to make causal conclusions. Finally, this study focused only on the perceived fairness/ severity of the penalty, which might be contaminated with other aspects of the penalty perception, as mentioned in the theory of deterrence. Although other studies have also used a similar approach, future studies might aim to include more variables related to traffic sanctions and the perceptions of justice.

## Conclusion

In general, Lithuanian offenders and non-offenders most frequently perceived the penalties for traffic rule offences as adequate and fair. Enforcement for drink driving was the offence most often evaluated as fair, when compared to the sanctions for speeding, aberrant driving, illegal phone use or other traffic violations. This implies that there is a need of education-prevention program of how serious and dangerous speeding, aberrant driving, illegal phone use or other traffic violations could be not only for society, but individually. Despite the fact that penalties for drink driving were evaluated as adequate, punished drivers or offenders with driving license suspension more often, than offenders who were sanctioned with monetary fine, perceived the penalty for driving under the influence of alcohol and the penalty for aberrant driving as too severe. Penalties for speeding violations were more often evaluated as too severe in the offenders’ group, whereas non-offenders more often evaluated these penalties as too mild. So, specialist who work with offenders rehabilitation courses need to have knowledge how to deal with defensiveness and adverse behavior caused by the feeling of injustice. Although differences in the perceptions of justice were not large, socio-demographic characteristics and the experience of traffic sanctions were related to the justice beliefs of specific penalties.

Funding for this research was received from the Research Council of Lithuania (LMTLT), agreement No. S-GEV-21-1.
